# Bilateral Lisfranc Injury in a Young Female: A Case Report

**DOI:** 10.7759/cureus.25238

**Published:** 2022-05-23

**Authors:** Hamid T ALJohani, Rheema Alfadhil, Lina Ismael, Sarah O Alturaisi, M Zahir F Aldalati, Abdulaziz Alahaideb

**Affiliations:** 1 Orthopaedic Surgery, King Saud Medical City, Riyadh, SAU; 2 Orthopaedic Surgery, King Saud University, Riyadh, SAU; 3 Family and Community Medicine, College of Medicine, King Saud University, Riyadh, SAU; 4 Nursing, King Saud Medical City, Riyadh, SAU

**Keywords:** bilateral tarsometatarsal fracture dislocations, lisfranc fracture dislocations, adult lisfranc fracture, bilateral lisfranc fracture, foot injuries

## Abstract

Lisfranc injuries are relatively rare, accounting for only 0.2% of all injuries. It is even rarer to have this injury bilaterally, and not many case reports have been published on this topic. In this report, we present a case of a bilateral Lisfranc injury in a healthy 17-year-old woman that fell from a flight of stairs landing on both feet. The diagnosis was made by weight-bearing x-rays and computed tomography. Weight-bearing x-rays showed a subtle Lisfranc injury in the right foot with widening between the first and second rays and a disruption involving the overlapping bases of the lesser metatarsals as well as a left comminuted fracture of the proximal third and fourth metatarsals (MT) with intra-articular extension at the proximal fourth MT. CT scan of the right foot showed a fracture of the lateral margin of the medial cuneiform with a displaced bony fragment as well as a comminuted fracture of the third and fourth metatarsals with intra-articular extension and no dislocation. Surgical management, in the form of open reduction and internal fixation, was undertaken for both feet in the same setting. She had an expected course postoperatively and has been doing well, with no pain nor limitation in her activity at her six-month postoperative visit. Moreover, we present a brief review of similar published cases and an overview of Lisfranc injuries.

## Introduction

Lisfranc injury is a term that describes any injury to the tarsometatarsal joint (TMTJ) complex that varies from simple ligamentous subluxation to devastating unstable open fractures of the bony structures [1]. Occurrences of TMTJ injuries are rare, accounting for about 0.2% of all fractures; some authors attribute that to the misdiagnosed cases reaching almost 35% as it is commonly overlooked, especially in polytrauma patients [2,3]. It is even rarer to have a bilateral presentation with only a few case reports in the literature and no randomized controlled trials or retrospective studies on this topic published to date [4]. The TMTJ injuries can be caused by direct or indirect forces [3]. The former is usually resulting from crush injuries to the foot, whereas the latter is due to overloading of a plantarflexed foot or forcible abduction of the forefoot [3]. Diagnosing this injury may be challenging as 11% of these injuries are subtle and are not easily picked up on standard radiographs [3]. Radiological clues can be aided by clinical examination findings, including localized tenderness alongside positive rotational and stress tests [3]. In this case report, we present a case report of a bilateral Lisfranc injury in a young woman.

## Case presentation

A 17-year-old woman, known to have bronchial asthma and iron deficiency anemia, presented to the emergency department in the December of 2020 after falling down several steps of stairs and landing on both feet. Her ankles were dorsiflexed, and she reported no twisting of the ankle joint. She was able to bear weight but with pain. Upon examining her feet, they were noticeably swollen at the medial dorsal area, and she had tenderness over the medial hindfoot while passively flexing the joint. She was able to perform minimal active range of motion but with pain. Despite the swelling, both feet showed a positive wrinkle test, indicating that she was fit for operative intervention at the time.

**Figure 1:**
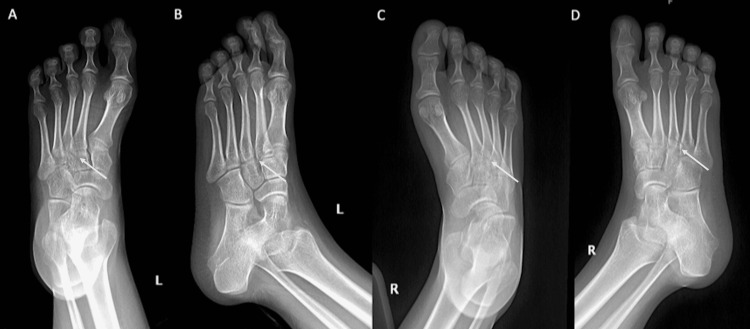
Anteroposterior and oblique x-ray views of both feet at the time of injury (A) Left foot AP x-ray. (B) Left foot oblique x-ray. (C) Right foot AP x-ray. (D) Right foot oblique x-ray. AP: Anteroposterior.

Weight-bearing x-rays were obtained for both feet, and they showed a subtle Lisfranc injury in the right foot with widening between the first and second rays and a disruption involving the overlapping bases of the lesser metatarsals as well as a left Lisfranc injury with a left comminuted fracture of the proximal third and fourth metatarsals (MT) with intra-articular extension at the proximal fourth MT (see Figure 1).

**Figure 2:**
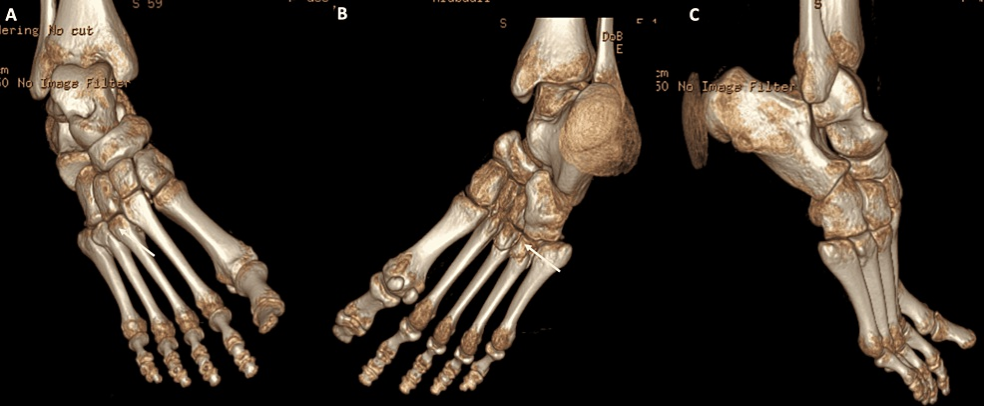
A 3D-reconstructed image of the computed tomography (CT) scan that was done to the right foot (A) Dorsal surface of the right foot. (B) Plantar surface of the right foot. (C) Lateral aspect of the right foot.

Unfortunately, no true lateral views were obtained at the time of injury. Because of the subtle injury in her right foot, we obtained a computed tomography (CT) image of the right foot only as the left foot showed a clear Lisfranc injury. The CT showed a fracture of the lateral margin of the medial cuneiform with a displaced bony fragment as well as a comminuted fracture of the third and fourth metatarsals with intra-articular extension and no dislocation (See Figure 2). Magnetic resonance imaging (MRI) was not ordered as there were bilaterally evident bony Lisfranc midfoot injuries. In addition, there was a concern about the progression of swelling with delaying the procedure until an MRI was obtained. 

Both feet were placed in backslaps for pain relief and to avoid further displacement and swelling. The decision was made to treat this patient operatively for both feet in the same setting under general anesthesia. For the right foot, she underwent open reduction with internal fixation and cannulated screws the following morning. A longitudinal incision was made over the TMTJ between the first and second MT, and dissection was carried out by layers until the joint was exposed.

**Figure 3:**
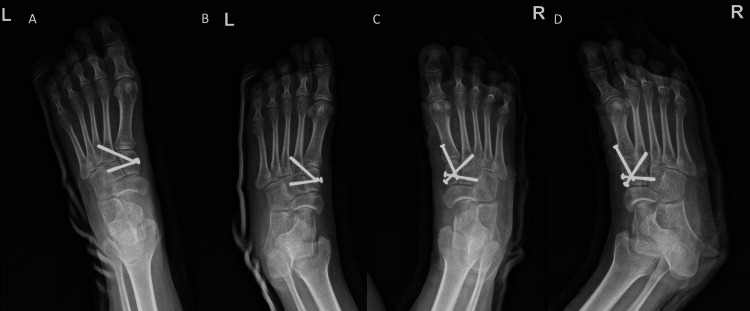
Immediate postoperative anteroposterior and oblique x-ray images of both feet (A) Left foot AP x-ray. (B) Left foot oblique x-ray. (C) Right foot AP x-ray. (D) Right foot oblique x-ray. AP: Anteroposterior.

Once the reduction was achieved and confirmed visually under x-rays (C-arm), the medial and middle cuneiform bones were fixed first, followed by first MT and medial cuneiform, then second MT with medial cuneiform fixation. All reductions were confirmed under the C-arm. Skin closure was done by layers, and the skin was closed with monocryl. A below-knee backslab was then applied. For the left foot, she underwent closed reduction and percutaneous screw fixation the following morning. The reduction was done and confirmed under C-arm. The medial and middle cuneiforms were fixed first, followed by fixation of the medial cuneiform with the base of the second MT. A below-knee backslab was applied. The surgery was successful, and there were no complications during the operation. Postoperative x-rays showed acceptable results (see Figure 3).

**Figure 4:**
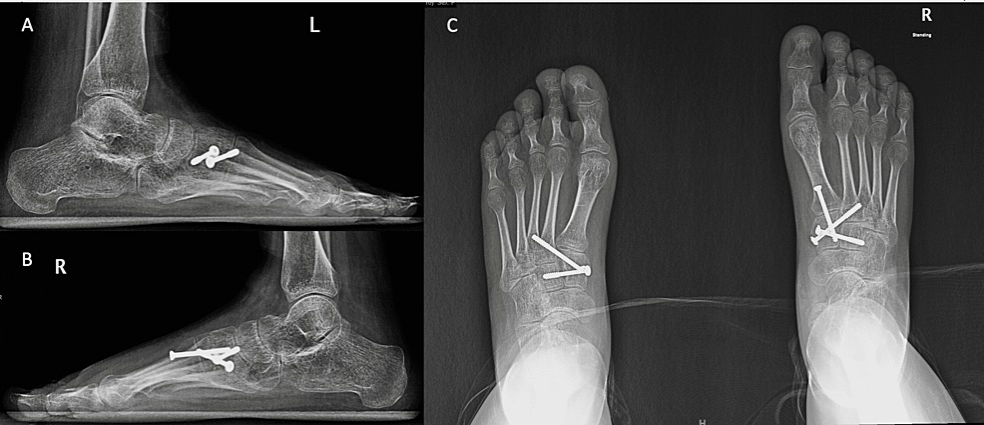
Weight-bearing anteroposterior and lateral x-ray images of both feet at postoperative six months (A) Weight-bearing left foot AP x-ray. (B) Weight-bearing left foot lateral x-ray. (C) Bilateral weight-bearing foot AP x-ray. AP: Anteroposterior.

From day one postoperatively until week six, she was mobilizing with physical therapy using the bed to chair mobilization, and then from week six until week eight, she was mobilizing on partial weight-bearing. After the eighth week, she started mobilizing full weight-bearing. The patient is still being followed in our clinic and could fully bear weight on both feet free of pain and with no limitations, which was evident in her latest visit that was approximately six months postoperatively (see Figure 4).

## Discussion

Lisfranc injuries involve a broad spectrum of TMTJ complex injuries ranging from ligament subluxation to unstable fractures [1]. Osseous and ligamentous structures provide stability to the midfoot. Injuries to these structures may cause instability and progress to a displacement of the tarsometatarsal joints [5]. These injuries can be caused by either direct or indirect forces [3]. The former results from crush injuries to the foot, whereas the latter occurs as a result of forcible abduction of the forefoot or overloading a plantarflexed foot [3]. 

Diagnosis of Lisfranc injuries can be difficult as conventional radiographs of the foot will not show ligament subluxation nor subtle injuries, and as a result, treatment may be delayed, and the patient may suffer from prolonged pain, post-traumatic arthritis, and flat foot [6-8]. The sensitivity of conventional radiographs is only 84% [7]. CT and MRI are some of the modalities that are used to assess TMTJ, which are superior to radiographs. CT scans can show uninterrupted images, and MRI is the gold standard modality as it visualizes soft tissues and ligamentous injuries [6,9]. It is documented that MRI has a sensitivity of 90% compared to intraoperative findings [7]. Choosing a treatment option depends on the severity and the level of displacement of the injury.

Radiological clues can be aided by clinical examination findings. These may include midfoot swelling, plantar ecchymosis, localized tenderness, positive rotational and stress tests, positive piano key test, and pain or instability with passive abduction of the midfoot while stabilizing the transverse tarsal joint [3,10]. The piano key test is performed by moving the head of the affected metatarsal while holding the midfoot firmly, which can help in isolating the exact TMTJ affected [10]. In patients who are able to bear weight, such as our patient, midfoot stability can be assessed by having the patient attempt a single-limb toe rise [10].

Non-displaced injuries on weight-bearing x-rays are typically treated non-operatively by a non-weight-bearing below-knee cast [7,10,11]. Open or displaced fractures are treated by either open reduction and internal fixation (ORIF) or closed reduction with percutaneous pinning or primary arthrodesis [5,7,8,12].

Carter and Wilby described a case of a 64-year-old woman with a bilateral Lisfranc injury after a motor vehicle accident. She underwent bilateral closed reduction and fixation using Kirschner wires (K-wires) [2]. Tadros and Al-Hossona [3] reported a case of a bilateral subtle TMTJ injury that was initially missed, which was sustained after a work-related injury in a 25-year-old man. His diagnosis was confirmed with a CT scan and was treated with closed reduction of both TMTJ with percutaneous screw fixation. He had an associated left calcaneus anterior process fracture that was treated with a small incision and fixed with a K-wire [3]. Vajapey and Miller [4] reported a similar case of a bilateral Lisfranc injury in a 17-year-old high-school athlete. He was treated with ORIF of the Lisfranc joint complex with an Arthrex TightRope fixation. At one year postoperatively, he returned to his full function, and his x-rays showed complete healing of the TMTJ and no residual diastasis [4].

There still remains a debate between ORIF and primary arthrodesis, a method of treating Lisfranc injuries [10]. Due to such contradiction and the presence of advantages and disadvantages to both treatment modalities, it is now currently left to the discretion of the primary surgeon. However, it is recommended to proceed with arthrodesis when a Lisfranc injury has been missed for greater than six weeks [10].

In our patient’s case, we decided to proceed with operative management in the form of open reduction with internal fixation and cannulated screws for the right foot and closed reduction and percutaneous screws fixation for the left foot. The surgery went well with excellent results. At six months postoperatively, the patient was back to her baseline activity level with neither limitation in activity nor pain.

Our current study has several limitations. First, it is a case report, which is the lowest form of evidence. Second, pre-operative lateral x-ray images were not available, and no MRI was obtained to further aid in the diagnosis. Third, our patient needs further follow-up to determine her long-term outcome.

## Conclusions

In this study, we reported a rather unlikely injury, wherein a healthy young woman developed a bilateral Lisfranc injury, and her both feet were treated surgically in a single setting. Moreover, similar cases described in the literature were mentioned, alongside some of the relevant available literature to provide an overview of Lisfranc injuries. To conclude, due to the persistent debate between ORIF and primary arthrodesis, we would recommend further studies on this matter, and for now, to leave it to the discretion of the primary surgeon.
